# Green Extraction and Targeted LC-MS Analysis of Biopesticides in Honey Using Natural Deep Eutectic Solvents

**DOI:** 10.3390/foods14193438

**Published:** 2025-10-08

**Authors:** Theaveraj Ravi, Alba Reyes-Ávila, Laura Carbonell-Rozas, Asiah Nusaibah Masri, Antonia Garrido Frenich, Roberto Romero-González

**Affiliations:** 1 Faculty of Chemical and Energy Engineering, Universiti Teknologi Malaysia, Johor Bahru 81200, Malaysia; rktheaveraj@gmail.com (T.R.); nusaibah@utm.my (A.N.M.); 2 Research Group “Analytical Chemistry of Contaminants”, Department of Chemistry and Physics, Research Centre for Mediterranean Intensive Agrosystems and Agri-Food Biotechnology (CIAIMBITAL), University of Almeria, Agrifood Campus of International Excellence, ceiA3, 04120 Almeria, Spain; ara494@ual.es (A.R.-Á.); lauracr@ual.es (L.C.-R.); agarrido@ual.es (A.G.F.)

**Keywords:** natural deep eutectic solvents, biopesticides, solid–liquid extraction, LC–HRMS analysis

## Abstract

Natural Deep Eutectic Solvents (NADES) were synthesized from food-grade components and evaluated as green extractants for the simultaneous recovery and liquid chromatography coupled to quadrupole-Orbitrap mass spectrometry (LC–Q-Orbitrap-MS) analysis of biopesticide residues in a complex matrix like honey. Conventional solid–liquid extraction (SLE) was applied, initially using choline chloride-2,3-butanediol (1:4, molar ratio) as the NADES extractant solvent, before systematically evaluating other NADES formulations. Extraction parameters, such as time (10 min, 20 min, and 30 min), technique (rotary mixing vs. sonication), and NADES composition, namely lactic acid–glucose–water (LGH, 5:1:9, molar ratio), lactic acid–glycerol–water (LGLH, 1:1:3, molar ratio), urea–glycerol–water (UGLH, 1:1:2, molar ratio), and choline chloride–2,3-butanediol (ChClBt, 1:4, molar ratio), were systematically optimized. Rotating agitation for 10 min yielded the highest overall recoveries and was therefore selected as the optimal extraction time. Rotary shaking was chosen over sonication due to its superior performance across both simple and complex matrices. Among the NADES tested, UGLH proved to be the most effective composition for the honey matrix. The analytical method was validated for the honey matrix. Linearity showed excellent performance across the tested concentration range, with R^2^ values above 0.95 for all analytes. Matrix effects were within ±20% for nearly half of the compounds, while a few exhibited moderate matrix enhancement. Recoveries ranged from 50.1% to 120.5% at 500 µg/kg and 1000 µg/kg, demonstrating acceptable extraction performance. Intra-day and inter-day precision showed relative standard deviations (RSDs) below 20% for most analytes. Limits of quantification (LOQs) were established at 500 µg/kg for eight compounds based on recovery and precision criteria. These results confirm the suitability of the proposed NADES-based method for sensitive and reliable analysis of biopesticide residues in honey. When compared to conventional extraction methods, the proposed NADES-based protocol proved to be a greener alternative, achieving the highest AGREEprep score due to its use of non-toxic solvents, lower waste generation, and overall sustainability.

## 1. Introduction

Pesticides are extensively used in agriculture to control pests, weeds, and diseases, significantly increasing crop yields and ensuring food security. Without them, fruit, vegetable, and cereal production would decrease by 78%, 54%, and 32%, respectively [1]. Pesticides, particularly synthetic ones, have significant negative impacts on both the environment and human health. Environmentally, they contribute to soil, water, and air pollution, impacting non-target organisms, such as beneficial insects, birds, and aquatic life. This can lead to a loss of biodiversity and disrupt the natural ecological balance [2]. On the human health front, exposure to pesticides has been linked to various serious health problems, including cancer, neurological disorders, reproductive issues, and even diabetes [1]. Infants and young children are especially vulnerable to pesticide exposure, making it a critical concern for public health [3].

These risks highlight the urgent need for safer and more sustainable alternatives, such as biopesticides. Biopesticides, derived from natural sources such as plants, animals, and microorganisms, include microbial pesticides, plant-incorporated protectants and biochemical pesticides [4]. They are widely used in organic farming and play a key role in Integrated Pest Management (IPM) programs [5]. Biopesticides are biodegradable, less toxic, and pose fewer risks to non-target organisms, helping to reduce soil and water pollution [2]. They are also considered safer for human health compared to synthetic pesticides [5]. Additionally, they can enhance crop yield and quality while being less likely to lead to pest resistance, making them a valuable tool in sustainable agriculture [1].

Despite being considered safer alternatives to synthetic pesticides, bioinsecticides can still pose risks to honey bee populations. Biopesticides can cause sublethal effects such as behavioral disruptions, developmental modifications and immune system impairments in honey bees [6]. For instance, exposure to certain biopesticides has been shown to affect honey bee foraging behavior and reduce their ability to pollinate effectively [7]. Some biopesticides can be lethal to honey bees, especially when used at high concentrations or in combination with other chemicals. For example, spinosad has been found to be highly toxic to honey bees, causing significant mortality even at low doses [8]. These bioinsecticides have been shown to negatively impact honey bee larvae, causing developmental issues and reduced survival rates [6]. Moreover, honey bees are often exposed to multiple stressors simultaneously, including biopesticides, synthetic pesticides, and environmental pollutants. These combined exposures can exacerbate the negative effects on bee health [7]. Given these risks, monitoring biopesticide residues in honey is critical, yet compared to synthetic pesticides, limited efforts have been devoted to developing sensitive and sustainable detection methods for this complex matrix [9].

For the analysis of biopesticides, the extraction has traditionally relied on conventional organic solvents such as methanol, acetonitrile, acetone, and *n*-hexane, which are effective in extracting bioactive compounds [10,11]. However, these solvents are flammable, toxic, and can lead to contamination of final products, raising safety and environmental concerns [2,12]. Consequently, there is a growing scientific interest in replacing conventional solvents with greener alternatives such as ionic liquids, deep eutectic solvents (DES) and supercritical fluids, which are being explored for their lower environmental impact and safety [13]. The development of environmentally friendly and sustainable extraction methods is essential, aligning with the inherent low toxicity and eco-friendly nature of biopesticides [11].

DES present a promising alternative to conventional extractants, especially in green sample preparation methods that involve complex food matrices. DES are biodegradable, non-toxic and exhibit selective extraction capabilities [14]. DES have been shown to effectively extract biopesticides from different matrices. DES have been successfully applied for the extraction of biopesticides, such as rotenone from Derris roots [15], as well as for pesticides including pyrethroids in tomatoes, organophosphorus compounds in honey and fruits, and herbicides, achieving recoveries between 55% and 113% [14,16,17]. Moreover, Natural Deep Eutectic Solvents (NADES), a subclass of DES formed from naturally derived components such as sugars, amino acids, or organic acids, have gained attention for their biocompatibility and sustainability [18]. The term “natural” refers to the use of bio-based, renewable, and food-grade substances, making NADES especially suitable for applications in food, agriculture, and pharmaceuticals due to their low toxicity and environmental compatibility [19].

However, to date, only a few studies have focused on the extraction of a single biopesticide, such as rotenone from the roots of *Derris elliptica* and *Derris malaccensis*, using alcohol-based NADES [15]. Very limited research has addressed the simultaneous extraction of multiple biopesticides from complex food matrices such as honey, where high sugar content and viscosity can hinder analyte recovery. This gap raises the question of whether NADES can be systematically optimized to achieve efficient and reproducible extraction of diverse biopesticide residues under realistic conditions. It is therefore hypothesized that NADES, owing to their tunable hydrogen-bonding interactions, polarity, and non-toxic nature, can provide both a sustainable and effective alternative to conventional solvents for the multi-residue analysis of biopesticides in honey. To test this hypothesis, four NADES composed of naturally derived components were synthesized and evaluated. The influence of solvent type, extraction time, and extraction technique was systematically investigated using LC-MS based on direct solid–liquid extraction (SLE), a green approach that eliminates the need for additional organic solvents while maintaining high analytical performance. The method was subsequently validated according to international guidelines and applied to real honey samples.

## 2. Materials and Methods

### 2.1. Materials

Analytical grade solvents, such as methanol (CH_3_OH, ≥99.9%), ethyl acetate (C_4_H_8_O_2_, ≥99.7%), and dodecanol (C_12_H_26_O, ≥99.7%), were provided from Honeywell (Charlotte, USA), whereas formic acid (CH_2_O_2_, 99.0%), and water (H_2_O, LiChrosolv^®^) were supplied from Merck (Darmstadt, Germany). All reagents for NADES synthesis were purchased from commercial suppliers: choline chloride (C_5_H_14_NO^+^Cl^−^, 67-48-1, ≥99.9%) from Fisher Scientific (Pittsburgh, PA, USA), lactic acid (C_3_H_6_O_3_, 50-21-5, ≥99.9%), glucose (C_6_H_12_O_6_, 50-99-7, ≥99.9%), and glycerol (C_3_H_8_O_3_, 56-81-5, ≥99.9%) from PanReac AppliChem (Barcelona, Spain), urea (CH_4_N_2_O, 57-13-6, ≥99.9%) from Sigma-Aldrich (St. Louis, MO, USA), and 2,3-butanediol (C_6_H_10_O_2_, 110-63-4, ≥99.9%) from Cymit Quimica (Barcelona, Spain).

Analytical standards of camphor were obtained from Alfa Aesar (Ward Hill, MA, USA); ricinine from Biosynth Carbosynth (Berkshire, UK); azadirachtin and α-solamargine from Chengdu Biopurify Phytochem (Chengdu, China); veratridine, cinnamyl alcohol, cinnamic acid, and cinnamaldehyde from Dr. Ehrenstorfer (Augsburg, Germany); tomatine from Extrasynthese (Genay, France); and cevadine from Phytolab (Vestenbergsgreuth, Germany). Additionally, acetyleugenol, pyrethrum extract (including cinerin I and II, pyrethrin I and II, and jasmolin I and II), pulegone, and nicotine were purchased from Sigma-Aldrich (St. Louis, MO, USA).

### 2.2. Analyzed Honey Samples

Seventeen honey samples were used in this study, consisting of both polyfloral and monofloral varieties. Honey sample 1 (polyfloral blossom from Spain, Cuba, Argentina and Ukraine) was used during the optimization of the extraction stage. The remaining 16 samples were used for method application and residue screening. These samples included polyfloral honeys of various floral compositions, such as wildflowers, albaida, and blends with eucalyptus and azahar, as well as monofloral honeys including azahar (orange blossom), thyme, lavender, rosemary, and heather. The samples were from diverse geographical regions, including Spain (Benahadux, Gádor, Granada, Lanjarón, Cataluña, Aragón, Galicia), Ukraine, Argentina, Chile, Mexico, Romania, Turkey, Vietnam, and Cuba. All honeys were obtained from local markets and commercial distributors, stored at room temperature, and analyzed without further treatment.

### 2.3. Synthesis of Natural Deep Eutectic Solvents (NADES)

NADES were synthesized by combining a hydrogen bond acceptor (HBA) and a hydrogen bond donor (HBD), and water when required, at approximately 60 °C until a homogeneous mixture was formed, following the stirring–heating method proposed by Dai et al. [20]. Typically, the component with the lowest melting point was melted first, followed by the gradual addition of the higher melting point component. The mixture was continuously heated until it was fully melted [14]. The following combinations were prepared with the indicated molar ratios: LGH (5:1:9), UGLH (1:1:2), ChClBt (1:4), and LGLH (1:1:3).

The evaluated NADES have been previously characterized in terms of their physicochemical properties, including pH, viscosity, density, and polarity [21,22]. The criteria for their preparation and selection in this study were guided by these properties, as they directly influence phase separation, analyte solubility, and extraction efficiency. A summary of the reported values is provided in Table S1 (Supplementary Materials).

### 2.4. Sample Preparation Procedure

For honey matrix, a direct extraction method using NADES was used. A sample of 1.00 g honey was mixed with 3 mL of the selected NADES, urea–glycerol–water (1:1:2, molar ratio), and vortexed for 10 min using a Vortex Mixer WX (Velp Scientifica, Usmate, Italy). After mixing, the samples were centrifuged at 7500 rpm (5590× *g*) for 10 min using a Centronic BL II centrifuge (JP Selecta, Barcelona, Spain). From the supernatant, a 500 μL aliquot was collected and diluted with 500 μL of methanol. The mixture was filtered through a 0.2 μm, 13 mm nylon syringe filter (Agilent Technologies, Santa Clara, CA, USA) and transferred into LC-MS vials for instrumental analysis.

### 2.5. UHPLC-Q-Orbitrap-MS Method

Chromatographic separation and detection were carried out using a Vanquish™ Flex Quaternary UHPLC system coupled to a Q-Exactive Orbitrap mass spectrometer (Thermo Fisher Scientific, Waltham, MA, USA), following the method developed by Reyes-Ávila et al. [11]. A Hypersil GOLD™ aQ (2.1 × 100 mm, 1.9 μm) Thermo Fisher Scientific column was used. The column oven temperature was maintained at 30 °C, with a flow rate of 0.2 mL/min and an injection volume of 10 μL. The mobile phase consisted of water with 0.1% formic acid (A) and methanol (B), using a gradient program as described previously [11]. Mass spectrometry detection was performed in full-scan mode with data-dependent acquisition (DDA) in both positive and negative ionization modes, as per the same reference. Instrument settings, including scan range, resolution, AGC targets, ionization parameters, and gas flow rates, were also described previously [11].

#### Data Analysis

All LC-MS data were acquired using the Xcalibur Sequence Setup software v4.1 (Thermo Fisher Scientific) and subsequently processed using Xcalibur 3.0, which includes the Qual and Quan Browser functionalities for qualitative and quantitative data analysis. A customized compound database was built using mzVault™ 2.3 SP1 and TraceFinder 4.0, enabling targeted screening of biopesticide residues. All software tools used were provided by Thermo Fisher Scientific. Mass accuracy was maintained within a 5 ppm error tolerance for all analyses.

### 2.6. Validation Process

The analytical method was validated for the honey matrix to ensure accuracy, reliability, and applicability of the extraction and detection protocols, according to the SANTE/11312/2021v2 guidance document [23]. Validation parameters included linearity, matrix effect, trueness (through recovery studies), precision, and limits of quantification (LOQs). For honey matrix, linearity was evaluated by spiking matrix samples at four concentration levels of biopesticides: 10, 50, 100, and 250 µg/L, each analyzed in triplicate. Calibration curves were built using matrix-matched standards, and recovery studies were performed at two spiking concentrations (500 µg/kg and 1000 µg/kg) using five replicates per level to assess method trueness, with average recoveries within the range of 70–120% deemed satisfactory. Intra- and inter-day precision were evaluated by analyzing five replicates at 500 and 1000 µg/kg on the same day, with relative standard deviation (RSD) values below 20% deemed satisfactory. The matrix effect was assessed by comparing signal intensities of analytes in matrix-matched standards to those in solvent-only standards, to evaluate ion suppression or enhancement during LC-Q-Orbitrap analysis. The acceptable range for matrix effects typically falls within ±20%.

## 3. Results and Discussion

### 3.1. Optimization of Biopesticide Extraction from Honey Matrix

A blank commercial honey sample (Honey 1), confirmed to be free of biopesticide residues, was spiked at 600 µg/kg with target biopesticides and used to optimize the extraction parameters and evaluate the performance of different NADES compositions. The extraction of biopesticides from honey matrix was studied using direct NADES-based extraction. The impact of three key parameters, including NADES type, extraction time, and extraction technique, on recovery efficiency was investigated across honey samples.

#### 3.1.1. Effect of Extraction Time

**Figure 1:**
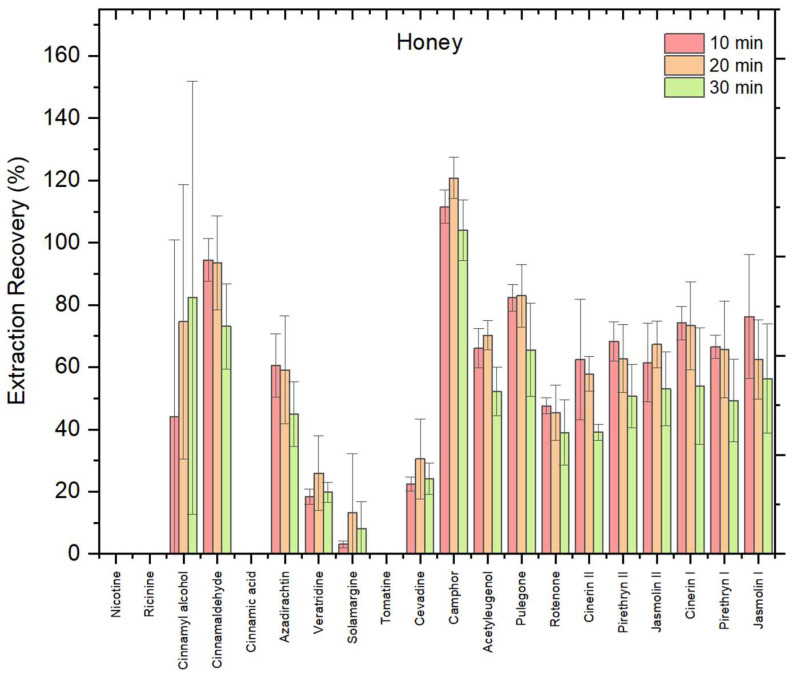
Recovery (%) of 20 biopesticides from honey matrix using ChClBt (1:4, molar ratio) at different extraction times (10, 20, and 30 min) in honey; error bars indicate relative standard deviation (*n* = 3).

To evaluate the influence of extraction time on analyte recovery from honey matrix, using ChClBt (1:4, molar ratio) as initial NADES, three extraction times were tested as follows: 10, 20, and 30 min. The reason why this NADES is chosen first is because choline chloride-based NADES, particularly when combined with polyols like butanediol, are among the most commonly used systems for extracting bioactive compounds from food matrices due to their effectiveness and versatility [24]. The results, shown in Figure 1, revealed that the recoveries across all time points were very similar for the honey matrix, with slight variations. Some biopesticides showed marginally higher recovery at 10 min, others at 20 or 30 min, indicating no significant improvement with longer extraction time. Overall, most compounds showed recoveries within an acceptable range (70–120%), including cinnamyl alcohol, cinnamaldehyde, camphor, pulegone, cinerin I, and jasmolin I. In contrast, certain compounds such as veratridine, solamargine, cevadine, and veratridine exhibited very low extraction efficiencies, with recoveries below 30%. Notably, some analytes such as nicotine, ricinine, cinnamic acid, and tomatine were not effectively extracted under tested conditions, confirming compound-dependent variability in NADES performance.

A one-way ANOVA confirmed that differences in mean recovery across the three extraction times were not statistically significant (F = 0.69, *p* = 0.54 > 0.05), indicating that extending extraction time beyond 10 min does not provide a significant analytical benefit. This is consistent with previous studies reporting that prolonged extraction in NADES does not necessarily improve recovery efficiency [22]. Considering both statistical results and analytical throughput, 10 min was selected as the optimal extraction time, ensuring efficiency without compromising method quality.

#### 3.1.2. Effect of NADES Type

**Figure 2:**
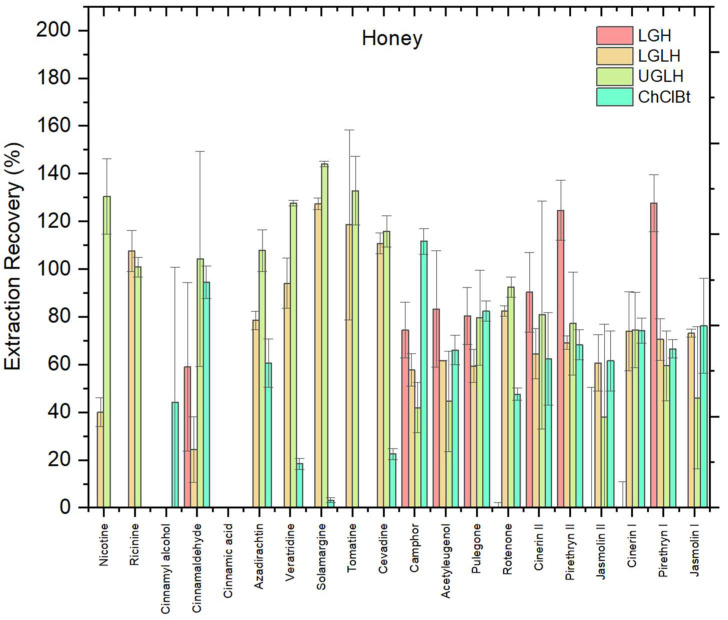
Recovery (%) of 20 biopesticides from honey matrix using four different NADES: LGH, LGLH, UGLH, and ChClBt in honey; error bars indicate relative standard deviation (*n* = 3).

The choice of NADES significantly influenced biopesticide recovery from the honey matrix. Four NADES were evaluated including LGH, LGLH, UGLH, and ChClBt. As shown in Figure 2, the optimal NADES order for biopesticides in honey was as follows: UGLH > LGLH > LGH > ChClBt. In our experiments, UGLH consistently showed better miscibility with all honey matrix compared to the other NADES. It dissolved more uniformly into the sample, creating a more stable and well-dispersed phase that likely enhanced the contact between the matrix and the extraction system. When evaluating the performance of UGLH for the extraction of biopesticides from honey, most of the compounds demonstrated recoveries within the acceptable range of 70–120%. This included nicotine, ricinine, cinnamaldehyde, azadirachtin, veratridine, solamargine, tomatine, cevadine, camphor, pulegone, rotenone, cinerin I, cinerin II, and pyrethrin II. In contrast, cinnamyl alcohol and cinnamic acid were not extracted under the tested conditions, confirming the limited applicability of UGLH for these analytes. Among the compounds that were extracted, none exhibited recoveries below 30%, suggesting that UGLH is generally effective for most of the tested biopesticides. The performance of UGLH may also be related to its near-neutral pH (8.9), as analyte extraction is generally more efficient when compounds remain stable and predominantly in their neutral form; similar trends have been observed in previous studies where recoveries improved around neutral pH compared to highly acidic or alkaline conditions [25].

This observation is supported by studies showing that urea and glycerol are both highly hydrophilic and water-soluble, which enables stronger hydrogen bonding with water and organic compounds in the matrix, promoting better mixing and analyte mobilization [26]. Additionally, UGLH has a relatively low viscosity, which improves phase dispersion and mass transfer during extraction [21]. These combined properties of high polarity compatibility, strong hydrogen bonding, and low viscosity explain the UGLH’s better interaction with the honey matrix compared to NADES based on lactic acid or choline chloride. In addition, the high performance of UGLH may be partly explained by its moderate viscosity and near-neutral pH, which favor efficient analyte partitioning and mass transfer, as reported in previous characterizations [21].

Moreover, a one-way ANOVA was performed to statistically evaluate the effect of NADES composition on overall recoveries. Although the analysis did not show statistically significant differences at the 95% confidence level (F = 3.10, *p* = 0.089 > 0.05), a clear trend was observed, with UGLH achieving the highest average recovery (79.9%) compared to LGH (54.0%), LGLH (68.7%), and ChClBt (59.2%). Beyond mean recoveries, the selection of UGLH was also supported by several qualitative criteria: this system provided more stable phase separation, sharper peak shapes, and stronger signal intensity compared to the other NADES formulations. Therefore, despite the lack of strict statistical significance, UGLH was selected as the optimal NADES for subsequent experiments.

#### 3.1.3. Effect of Extraction Technique

**Figure 3:**
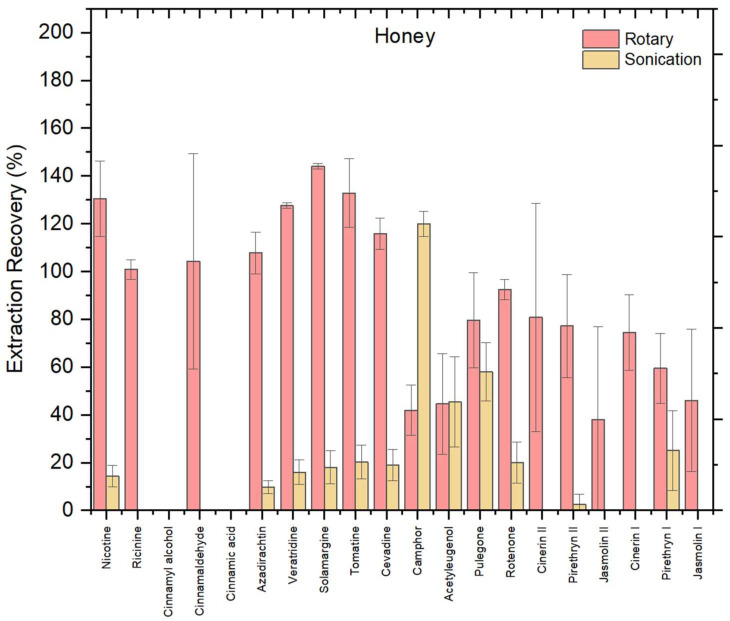
Recovery (%) of 20 biopesticides from honey matrix using the optimal UGLH, comparing rotary mixing and sonication in honey; error bars indicate relative standard deviation (*n* = 3).

To assess the impact of extraction technique on biopesticide recovery from the honey matrix, the optimized NADES (UGLH) was applied by comparing two methods: rotary mixing and sonication (Figure 3). Rotary mixing consistently yielded higher recoveries across honey matrix compared to sonication. Using the rotary method, most compounds exhibited recoveries within the acceptable 70–120% range, including nicotine, ricinine, cinnamaldehyde, azadirachtin, veratridine, solamargine, tomatine, cevadine, camphor, pulegone, rotenone, cinerin I, cinerin II, and pyrethrin II. None of the extracted compounds showed recoveries below 30%. However, cinnamyl alcohol and cinnamic acid were not extracted under rotary conditions, indicating limitations of the method for these compounds.

In contrast, sonication yielded notably weaker results, with a larger number of compounds not extracted at all. These included cinnamyl alcohol, cinnamic acid, ricinine, cinnamaldehyde, cinerin I, cinerin II, jasmolin I, and jasmolin II. The continuous and gentle agitation provided by rotary mixing enhances analyte–solvent interactions and promotes uniform contact between the NADES and the matrix [20]. In contrast, although sonication is known to speed up the release of compounds by disrupting matrix structures, it may inadvertently lead to localized heating or microcavitation, which can induce thermal degradation of sensitive biopesticides or reduce phase separation efficiency, particularly in viscous NADES systems [27].

A one-way ANOVA confirmed that the observed difference between rotary mixing and sonication was statistically significant (F = 36.2, *p* = 0.0038 < 0.05), supporting the selection of rotary mixing as the superior extraction technique. Beyond statistical significance, rotary mixing also offers greater operational simplicity, scalability, and compatibility with green chemistry principles.

Based on the optimization results, UGLH was selected as the solvent for the honey matrix, with a 10 min extraction time using rotary agitation. The optimized method was subsequently validated.

### 3.2. Validation of Biopesticide Extraction from Honey Matrix

To confirm the effectiveness and reliability of the NADES-based extraction method for analyzing biopesticide residues in honey, a comprehensive validation was conducted. The parameters assessed included (i) matrix effect, (ii) linearity, (iii) trueness, and (iv) precision (both intra-day and inter-day). However, only sixteen compounds were validated, evaluated across two concentration levels (500 µg/kg and 1000 µg/kg), as four biopesticides (cinnamyl alcohol, cinnamaldehyde, cinnamic acid, and camphor) showed incompatible results, with recoveries and reproducibility falling outside acceptable ranges. According to the SANTE/11312/2021v2 guidelines, analytes with recoveries outside the 70–120% range but with acceptable precision (RSDs ≤ 20%) can be corrected using a recovery correction factor [23]. However, compounds such as tomatine, which showed both low recovery (62.4%) and poor precision (RSD > 23%) for 1000 µg/kg, do not meet the SANTE acceptance criteria. For such analytes, correction factors cannot be applied, and they should be considered outside the validated scope of the method. Table 1 shows a summary of validation results of biopesticides in the honey sample.

#### 3.2.1. Matrix Effect

Matrix effect validation is a crucial step in the extraction and quantification of biopesticides from honey matrix. The matrix effect refers to the alteration in the analytical signal of a biopesticide due to the presence of other substances in the sample matrix, which can lead to either signal enhancement or suppression [28]. This phenomenon can significantly impact the accuracy and reliability of biopesticide residue analysis [29]. The acceptable range for matrix effects typically falls within ±20%. This range indicates that the matrix effect should not cause more than a 20% deviation in the analytical signal compared to a matrix-free standard [30].

Seven out of the 16 tested biopesticides showed matrix effects within the acceptable range of ±20%, including ricinine (−19.6%), jasmolin I (−9.9%), jasmolin II (−9.5%), solamargine (14%), tomatine (6.6%), rotenone (10.7%), and cinerin I (−9.9%). For the remaining compounds, matrix enhancement above 20% was observed, particularly for nicotine (36.2%), acetyleugenol (33.9%), pyrethrin II (40.4%), and pulegone (31.7%). Due to the presence of significant matrix effects in several analytes, matrix-matched calibration was employed to ensure accurate quantification.

#### 3.2.2. Linearity

All analytes showed strong linear correlation coefficients (R^2^), with 15 out of 16 compounds scoring above 0.98. Nicotine (0.999), ricinine (0.997), solamargine (0.998), and tomatine (0.998) demonstrated excellent linearity, confirming that signal response is proportionally related to analyte concentration. This linear behavior is essential for trace detection and quantification in residue analysis. Even the lowest R^2^ observed, acetyleugenol (0.978), remained within acceptable thresholds for semi-quantitative analysis. In addition, the deviation of back-calculated concentrations from the true values did not exceed ±20%, in agreement with the SANTE guidelines. These findings support the use of matrix-matched calibration curves to overcome the variability caused by matrix effects [31]. Overall, the high R^2^ values across all tested analytes confirm the method’s suitability for accurate and reliable quantification of biopesticides in complex food matrices like honey.

#### 3.2.3. Trueness

Recovery studies were carried out to evaluate the trueness of the NADES-based extraction method by determining how efficiently biopesticides could be extracted from the honey matrix. These tests were performed at two concentration levels 500 µg/kg and 1000 µg/kg, using five replicates for each point. The majority of biopesticides showed recoveries within the generally accepted range of 70% to 120%, confirming the method’s capability to extract analytes reliably from a complex sugar-rich matrix like honey.

Compounds such as ricinine, cevadine, and pyrethrin II showed excellent recovery profiles across both concentration levels, often exceeding 90%, suggesting high solubility in the NADES system and effective mass transfer during extraction. Furthermore, it shows that NADES effectively facilitated analyte extraction from the honey matrix. The consistency across both concentration levels further supports the method’s applicability for semi-quantitative analysis in food residue monitoring. A few biopesticides, however, presented lower recovery values, notably jasmolin I, tomatine, and acetyleugenol, with recoveries falling below 70% at one or both concentration levels. This could be attributed to factors such as limited solubility in the selected NADES composition, polarity between NADES and biopesticides, or partial degradation during the extraction process [32].

The application of correction factors for analytes with recoveries <70% should be considered with caution. While SANTE/11312/2021v2 permits their use when results are reproducible, this approach may introduce additional uncertainty and reduce the reliability of quantification [23]. In this study, correction factors were only applied when recovery values were consistent and precision was acceptable (RSD < 20%). Nevertheless, these compounds highlight the limitations of the current NADES formulations, and future optimization or pre-concentration strategies may be required to improve extraction efficiency for more challenging analytes.

Beyond the procedural correction factors, the chemical basis for low recoveries should also be considered. One important aspect is polarity mismatch, where nonpolar analytes tend to partition poorly into more polar NADES formulations, consistent with reports that NADES polarity strongly affects solubility and extraction efficiency [33]. In addition, some biopesticides may undergo instability or partial degradation during extraction, leading to reduced recovery [34]. Finally, the complex honey matrix (rich in sugars and other co-extractives) can interfere with analyte partitioning and cause ion suppression in LC–MS, further contributing to poor recovery [35]. These factors together explain the reduced recoveries of compounds and highlight the need for polarity tuning and pre-treatment strategies in future NADES studies.

Nonetheless, the use of five replicates per level improved statistical validation and confirmed that the observed recovery trends were consistent. Given the good repeatability observed for acetyleugenol, azadirachtin, and pyrethrin I (see Table 1), with RSD values below 20% and recoveries exceeding 50%, the application of a correction factor is considered acceptable. Overall, the method demonstrated acceptable accuracy for most target compounds and showed promise for further application in the quantitative analysis of biopesticide residues in honey.

#### 3.2.4. Precision

Precision was evaluated in terms of both intra-day (repeatability) and inter-day (reproducibility) performance to assess the method’s stability over time. In pesticide residue analysis, RSD values below 20% are generally considered acceptable for intra-day precision, indicating reliable and consistent measurements within the same analytical run [36]. Most biopesticides displayed excellent intra-day precision, with RSDs well below 20%. Ricinine (7.7–9.6%), rotenone (6.5–11.1%), cevadine (8.7–10.6%) and pyrethrin I (7.3–8.0%) exemplified highly stable results, confirming the method’s consistency in repeated measurements and minimal variation due to random analytical errors or matrix complexity. Nonetheless, a few compounds presented higher intra-day RSDs, suggesting possible instability or stronger interactions with the honey matrix. For instance, tomatine (23.3%) demonstrated relatively poor reproducibility within a single batch of analysis. Some biopesticides may be chemically unstable, leading to degradation or transformation during analysis. This instability can result in inconsistent measurements, contributing to higher RSD values [37]. While 15 out of the 16 compounds met the precision criterion, care should be taken with more unstable analytes by optimizing extraction time or storage conditions to minimize degradation.

Inter-day precision reflects the method’s reproducibility across different days, accounting for variations in ambient conditions, analyst handling, and equipment fluctuations [38]. The majority of the compounds demonstrated satisfactory inter-day reproducibility, with RSD values under 20%. For instance, azadirachtin and rotenone showed consistent results with RSDs ranging from 7.3% to 10.1%, while cevadine, veratridine, pyrethrin I, and pulegone were also within acceptable reproducibility limits. This consistency highlights the method’s validity and operational stability over time, particularly when working with a complex food matrix like honey. However, only one biopesticide, jasmolin I (21.5%), exceeded the acceptable RSD threshold for inter-day variation at 500 µg/kg, possibly due to analyte instability, degradation, or inconsistent recovery influenced by matrix interactions [39]. Despite these outliers, the method showed acceptable precision for 15 out of 16 biopesticides, affirming its general suitability for routine multi-day monitoring.

#### 3.2.5. Limit of Quantification (LOQ)

LOQ represents the lowest concentration of an analyte that can be quantitatively detected with acceptable precision and trueness under validated conditions [40]. In this study, LOQs were determined based on the lowest level of spiking tested (500 µg/kg) that fulfilled the SANTE/11312/2021 criteria, specifically, recoveries within 70–120% and relative standard deviation (RSD) values below 20% [23]. Among the sixteen biopesticides tested, eight compounds (cevadine, jasmoline II, pulegone, pyrethrin II, ricinine, rotenone, solamargine, and veratridine) met these criteria at the 500 µg/kg level and thus this concentration was established as their LOQ.

**Figure 4:**
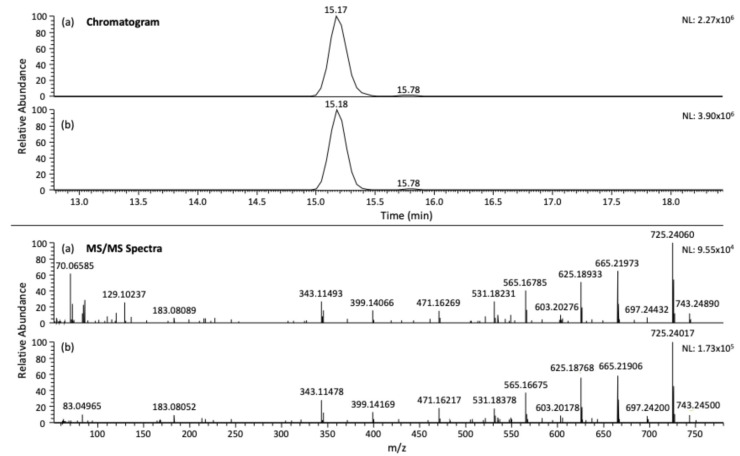
UHPLC-Q-Orbitrap chromatogram and MS/MS spectra of (**a**) standard of azadirachtin at 500 µg/L and (**b**) azadirachtin (500 µg/kg) after extraction with UGLH (1:1:2, molar ratio).

For the remaining compounds, the validated LOQ was assigned as 1000 µg/kg for azadirachtin, cinerin I, and cinerin II, whereas acetyleugenol, nicotine, pyrethrin I, jasmolin I, and tomatine did not meet acceptance criteria even at 1000 µg/kg, indicating that their LOQs lie at this level under the current method conditions. While this dataset did not include lower spiking levels, these results suggest that further refinement such as evaluating additional lower concentrations or performing a signal-to-noise (S/N) assessment using LC–Q-Orbitrap software Xcalibur v4.1 would be necessary to establish more precise LOQs for those compounds [41]. Nevertheless, the method demonstrated sufficient sensitivity for a subset of biopesticides, supporting its utility for monitoring residues in complex food matrices such as honey. As can be seen in Figure 4, the chromatogram and mass spectrum of the azadirachtin standard (a) were identical to those obtained in matrix after validation, (b) confirming the accurate identification of the compound.

Currently, regulatory maximum residue levels (MRLs) have been established for only a limited number of pesticides in honey. For example, the existing EU MRL for acetamiprid in honey was initially set at the limit of quantification (0.05 mg/kg) but was recently re-evaluated by EFSA, with proposals to raise it up to 1 mg/kg based on residue data from semi-field trials (EFSA, 2025). Analytical enforcement methods for honey are typically validated with LOQs of 0.01–0.05 mg/kg [41]. In contrast, the LOQs achieved in this study (500–1000 µg/kg) are considerably higher. This confirms that while the NADES-based method demonstrates proof-of-concept for green extraction of biopesticides, further sensitivity improvements are required before it can be applied to regulatory monitoring of honey.

### 3.3. Application to Real Honey Samples

The validated method was applied to the analysis of seventeen commercial honey samples, consisting of both polyfloral and monofloral varieties collected from various geographic regions. Honey sample 1 was used for method optimization and the remaining sixteen were screened for biopesticide residues under the established extraction and detection conditions. No detectable residues of the targeted biopesticides were found in any of the seventeen honey samples. These findings suggest either the absence of contamination or the presence of residues below the method’s LOQ [41]. The results support the applicability of the developed NADES-based method for reliable screening of biopesticide residues in real honey matrices.

### 3.4. Green Assessment and Comparison with Conventional Methods

The performance and environmental impact of the developed NADES-based extraction protocol were evaluated and compared with conventional sample preparation techniques commonly applied for pesticide and biopesticide analysis in honey, including quick, easy, cheap, effective, rugged, and safe (QuEChERS), restricted access materials (RAM) combined to molecularly imprinted polymers (MIPs) and liquid–liquid extraction (LLE) [42,43,44].

The NADES method demonstrated recoveries ranging from 50.1% to 120.5% for 16 biopesticides at two concentration levels (500 and 1000 µg/kg), with intra- and inter-day RSD values mostly below 20%, meeting international validation criteria. However, the method LOQs were relatively high (500–1000 µg/kg), reflecting the complexity of the honey matrix. This performance is comparable to that of He et al., who used the RAM-MISPE method for the extraction of six organophosphorus pesticides (malathion, ethoprophos, phorate, terbufos, dimethoate, and fenamiphos) from honey, achieving recoveries between 89.2 and 97.8%, RSDs of 2.25–5.12%, and LOQs as low as 0.5–1.9 µg/kg, though their method involved complex preparation, sorbent packing, and the use of methanol and acetone as extractants [42]. Similarly, Santos et al. developed and validated a liquid–liquid extraction (LLE) method with low-temperature purification (LTP) for the determination of organochlorine pesticides (Aldrin and Mirex) in honey, achieving recoveries close to 100% with RSDs < 10% and LOQs of 2.0–4.0 µg/kg [43]. In comparison, the NADES-based approach employs no hazardous organic solvents, requires no sorbent cleanup and achieves satisfactory recoveries with simplified handling. The QuEChERS method reported good performance in honey, with recoveries of 70–120%, RSDs < 20%, and LOQs as low as 0.2–8 µg/kg across 209 analytes. However, it still involves large amounts of acetonitrile/ethyl acetate, salting-out agents, and solid-phase sorbents like PSA and GCB, resulting in greater chemical consumption and waste generation [44].

**Figure 5:**
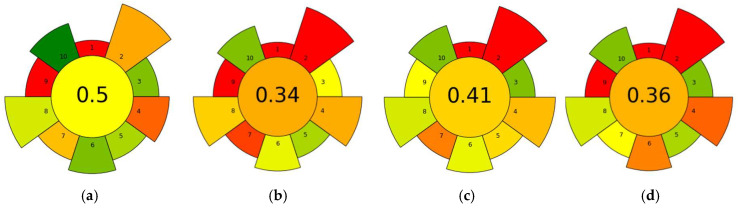
AGREEprep score for (**a**) NADES-based extraction method (this study), (**b**) QuEChERS method [44], (**c**) RAM-MISPE method [42], and (**d**) LLE method [43]. Pictograms showed the ten evaluation criteria (1–10) defined by the AGREEprep software. Colors represent the relative performance of each criterion, from green (more sustainable) to red (less sustainable).

To further evaluate the sustainability of the proposed extraction protocol, the AGREEprep tool was used to assess the greenness of sample preparation based on ten criteria aligned with the green sample preparation (GSP) principles [45]. The resulting radar chart (Figure 5) compares the NADES-based method with three conventional extraction techniques: QuEChERS, RAM-MISPE, and LLE, each applied to pesticide or biopesticide residue analysis in honey.

The AGREEprep score for the NADES-based method was 0.5, the highest among all evaluated methods. In comparison, RAM-MISPE scored 0.41, LLE 0.36, and QuEChERS 0.34. The higher score of the NADES method is largely attributed to its low usage of non-hazardous, non-toxic extractants, low solvent consumption, and reduced waste generation. In contrast, the conventional methods suffered from multiple “red” indicators due to the use of higher toxic or hazardous organic (criterion 2) solvents such as acetonitrile, methanol, and ethyl acetate, as well as multi-step workflows (criterion 7). Moreover, ex situ sample preparation (criterion 1) and post-sample preparation analysis using LC-MS or gas chromatography–mass spectrometry (GC-MS) (criterion 9) are common to all evaluated methods. However, as demonstrated in this study, the overall greenness can be significantly improved through the use of non-hazardous materials such as NADES.

## 4. Conclusions

This study successfully developed and validated a green sample preparation for the extraction and quantification of biopesticide residues in honey using NADES. Among the NADES formulations tested, the UGLH (1:1:2, molar ratio) system demonstrated the highest extraction efficiency for the honey matrix. Optimization of key parameters, including extraction time and mixing technique, further enhanced method performance. Moreover, this study demonstrates that NADES enable the simultaneous extraction of multiple biopesticides from honey, in contrast to previous studies that mainly focused on a single compound. This highlights the capability of NADES as efficient extractant solvents and their potential as a green and promising alternative to conventional organic solvents for residue analysis. Compared to conventional extraction techniques, the NADES-based method developed in this study achieved satisfactory recoveries for several analytes and offered a significantly greener alternative, as confirmed by AGREEprep assessment, although its LOQs were higher than those typically reported for conventional methods. These findings underscore the potential of NADES as sustainable alternatives to conventional solvents in residue analysis, though further optimization is needed before routine monitoring applications in complex food matrices such as honey can be realized. Nonetheless, further studies are needed to enhance sensitivity while increasing the number of compounds that can be quantified with acceptable recovery levels.

## Figures and Tables

**Table 1:** Summary of validation results of the biopesticides in the honey sample (*n* = 5).

Compound	R^2^	Matrix Effect (%)	Recovery			
			Intra-Day (%)		Inter-Day (%)	
			500 µg/kg	1000 µg/kg	500 µg/kg	1000 µg/kg
Acetyleugenol	0.978	+33.9	62.3 (7.5) ^1^	68.9 (19.8) ^1^	63.4 (18.9) ^1^	68.3 (19.1)
Azadirachtin	0.998	+24.0	69.1 (14.1) ^1^	81.6 (10.9)	70.3 (9.4)	87.6 (7.3)
Cevadine	0.998	+25.5	97.7 (8.7)	94.2 (10.6)	79.4 (12.6)	81.2 (19.5)
Cinerin I	0.989	−9.9	60.5 (9.2) ^1^	75.1 (10.1)	67.0 (19.8)	71.4 (14.9)
Cinerin II	0.992	+23.0	-	79.8 (18.5)	-	76.5 (15.7)
Jasmolin I	0.990	−9.9	50.1 (19.5) ^1^	60.6 (6.5) ^1^	55.4 (21.5)	68.4 (19.8) ^1^
Jasmolin II	0.990	−9.5	85.2 (18.9)	88.7 (19.5)	80.1 (16.8)	70.6 (19.9)
Nicotine	0.999	+36.2	-	67.9 (20.1)	-	84.4 (11.3)
Pulegone	0.998	+31.7	70.2 (15.3)	97.5 (7.3)	71.0 (18.7)	78.2 (9.5)
Pyrethrin I	0.994	+31.2	53.2 (8.0) ^1^	67.9 (7.3) ^1^	62.5 (18.3) ^1^	71.2 (3.0)
Pyrethrin II	0.985	+40.4	96.7 (8.4)	120.5 (9.6)	81.3 (19.5)	118.6 (10.4)
Ricinine	0.997	−19.6	106.6 (7.7)	97.1 (9.6)	87.1 (15.2)	90.1 (17.2)
Rotetone	0.994	+10.7	75.4 (6.5)	90.7 (11.1)	72.0 (10.1)	82.1 (8.8)
Solamargine	0.998	+14.0	82.7 (10.0)	71.8 (17.7)	72.2 (13.4)	84.9 (15.7)
Tomatine	0.998	+6.6	61.7 (15.0) ^1^	62.4 (23.3)	70.8 (15.3)	82.4 (20.0)
Veratridine	0.995	+27.8	82.0 (19.8)	89.9 (10.3)	85.3 (13.5)	87.4 (17.3)

^1^ Correction factor applied due to recovery <70% with acceptable RSD (≤20%), in accordance with SANTE/11312/2021.

## Data Availability

The original contributions presented in this study are included in the article. Further inquiries can be directed to the corresponding author.

## Supplementary Materials

The following supporting information can be downloaded at https://www.mdpi.com/article/10.3390/foods14193438/s1; Table S1. Physicochemical properties of the evaluated NADES [21,22].

## Abbreviations

The following abbreviations are used in this manuscript:

ChClBt	Choline Chloride–2,3-Butanediol
DDA	Data-Dependent Acquisition
HBA	Hydrogen Bond Acceptor
HBD	Hydrogen Bond Donor
IPM	Integrated Pest Management
LGH	Lactic acid–Glucose–Water
LGLH	Lactic acid–Glycerol–Water
LOQ	Limits of Quantification
NADES	Natural Deep Eutectic Solvent
RSD	Relative Standard Deviation
SLE	Solid–Liquid Extraction
UGLH	Urea–Glycerol–Water
