# Supplementary material for: Green Extraction and Targeted LC-MS Analysis of Biopesticides in Honey Using Natural Deep Eutectic Solvents
Source: Foods. 2025 Oct 8;14(19):3438. doi: 10.3390/foods14193438 (PMC12523273; doi:10.3390/foods14193438)
Supplement: Supplementary file 1 [file foods-14-03438-s001.zip › foods-3907346-supplementary.pdf]

Supplementary Materials:

Table S1. Physicochemical properties of the evaluated NADES [1, 2]

NADES	Comp. 1 (HBA)	Comp. 2 (HBD)	Comp. 3	Molar Ratio	pH	Viscosity (mPa*s)	Density (g/cm^3)	Polarity E_{NR}^{N} (kcal/mol)
LGH	Lactic acid	Glucose	Water	5:1:9	1.07	44	1.20	n.r.[a]
LGLH	Lactic acid	Glycerol	Water	1:1:3	1.40	26	1.18	1.086
UGLH	Lactic acid	Glycerol	Water	1:1:2	8.90	27	1.21	n.r.
ChClBt	Choline Chloride	2,3-Butanediol	-	1:4	4.6	n.r.	n.r.	0.771

[a] n.r. Not reported

References

1. Carbonell-Rozas, L., et al., *Structural characterization and physicochemical properties of different hydrophilic natural deep eutectic solvents.* Analytical and Bioanalytical Chemistry, 2025. **417**(1): p. 183-197.
2. Capilla-Flores, R., et al., *Natural deep eutectic solvent-based extraction for isolating non-phthalate plastic additives from radish samples (Raphanus sativus L.).* Sustainable Chemistry and Pharmacy, 2025. **47**: p. 102138.
